# Changes in intestinal microflora in digestive tract diseases during pregnancy

**DOI:** 10.1007/s00404-019-05336-0

**Published:** 2019-11-27

**Authors:** Min Jin, Dong Li, Rui Ji, Wen Liu, Xiaofei Xu, Yanqing Li

**Affiliations:** 1 Department of Anesthesiology, Qilu Hospital, Shandong University, Jinan, 250012 Shandong China; 2 Stem Cell and Regenerative Medicine Center of Shandong University, Jinan, 250012 Shandong China; 3 Department of Gastroenterology, Qilu Hospital, Shandong University, Jinan, 250012 Shandong China; 4 Department of Obstetrics and Gynecology, Center for Reproductive Medicine, Qilu Hospital, Shandong University, Jinan, 250012 Shandong China; 5 Department of Gastroenterology, Laboratory of Translational Gastroenterology, Qilu Hospital, Shandong University, Jizhong Building, 107 Wen hua Xi Road, Lixia District, Jinan, 250012 Shandong China

**Keywords:** Digestive diseases, Pregnancy, Intestinal microflora, 16 s rRNA sequencing

## Abstract

**Purpose:**

This study aimed to investigate the gut microbiome composition in pregnant women with digestive diseases to analyze the relationships between the microflora changes and digestive diseases during pregnancy.

**Methods:**

Fecal samples obtained from 71 pregnant women [six acute fatty liver (AF group), 21 constipation (C group), 24 excessive vomiting (V group) and 20 normal pregnancy (CP group)] and 26 non-pregnant (NP group) women were subjected to 16 s rRNA sequencing. Differential analysis of intestinal flora at the genera level was performed.

**Results:**

The relative abundance of *Coprobacillus*, *Acinetobacter*, *Enterococcus*, *Weissella* and *Lysinibacillus* was increased in the digestive diseases (AF, C and V) groups compared with CP group, whereas that of five common genera, including *Terrisporobacter*, *Dysgonomonas*, *Adlercreutzia*, *Fusicatenibacter* and *Blautia*, was decreased in digestive diseases groups. Additionally, in digestive diseases (AF, C and V) groups, the abundance of 13 common genera, such as *Carnobacterium*, *Coprobacillus* and *Psychrobacter*, was higher than NP group, whereas that of 27 common genera, such as *Blautia* and *Terrisporobacter*, was lower than NP group. About 69 genera were differentially abundant between AF and C groups; two genera (*Aerococcus* and *Senegalimassilia*) were identified between AF and V groups; moreover, total 63 genera were obtained between C and V groups.

**Conclusion:**

Our data revealed that the abundance of *Acinetobacter*, *Enterococci*, *Paenibacillus*, *Blautia* and *Collinsella* might be associated with the digestive diseases during pregnancy. These findings further supported the idea that targeting the gut microbiota could be a new prevention or therapeutic approach for improving digestive diseases during pregnancy.

**Electronic supplementary material:**

The online version of this article (10.1007/s00404-019-05336-0) contains supplementary material, which is available to authorized users.

## Introduction

During pregnancy, the pregnant woman usually undergoes significant physiological changes so as to accommodate and nurture the developing fetus, and these changes can result in a diversity of symptoms [1, 2]. It has been reported that pregnancy is a challenging period for the gastroenterologist, and multiple gastrointestinal complaints such as nausea, vomiting, gastroesophageal reflux, heartburn and constipation may occur [3]. The prevalence of nausea ranges from 50 to 80% and of vomiting is 50% [4]. Hyperemesis gravidarum is a severe form of nausea and vomiting, which affects about 1.2% of pregnant women [5].

In addition to gastrointestinal disorders, pregnancy is also associated with increased liver metabolism, and 3–5% of pregnancies present abnormal liver functions [6]. Liver disease during pregnancy is non-negligible, which may be life threatening for mother, fetus and subsequently the surviving child [7]. Acute fatty liver of pregnancy is an uncommon liver disease that occurs almost exclusively in the third trimester [8]. The reported incidence values for this disease are 1:7000–15,000 pregnancies [9, 10]. Acute fatty liver of pregnancy is a potentially fatal disease needing early diagnosis and intervention to prevent maternal and fetal mortality [6].

Growing evidences have shown that intestinal gut microbiota, a robust ecosystem inhabited by nearly 100 trillion bacteria, has an influence on intestinal physiological function and is involved in the life activities [11, 12]. For instance, chronic constipation, a prevalent functional gastrointestinal disorder, is demonstrated to be accompanied with intestinal dysbiosis [13]. Boursier et al. [14] recently reported that the severity of nonalcoholic fatty liver disease was implicated in gut dysbiosis and changes in metabolic function of the gut microbiota. However, there is presently little research about the changes in intestinal microbial communities in pregnant women with digestive diseases, and about how these changes affect the digestive system of patients.

In this study, we investigated the gut microbiome composition in fecal samples from pregnant women with digestive diseases, to analyze the relationships between the microflora changes and digestive diseases during pregnancy. The results may provide certain theoretical basis for safe, noninvasive diagnosis, treatment and intervention of digestive diseases during pregnancy.

## Materials and methods

### Sample collection

A total of 71 pregnant and 26 non-pregnant (NP group) women were involved in this study. Among the pregnant women, six cases had acute fatty liver (AF group), 21 cases had constipation (C group), 24 cases had excessive vomiting (V group) and 20 cases had normal pregnancy (CP group). The detailed information is listed in supplementary Table 1. Fecal samples were obtained from 97 women and stored at − 80 °C within 2 h.

**Table 1:** Alpha diversity indexes for all groups

Group	Observed_species	Shannon	Simpson	Chao1	ACE	Goods_coverage	PD_whole_tree
AF	2099	6.98	0.948	2843.739	2978.057	0.981	148.742
C	702	6.243	0.947	859.857	875.363	0.996	49.541
CP	809	5.688	0.918	1093.32	1143.516	0.993	60.163
NP	1209	6.005	0.905	1555.113	1617.687	0.99	91.423
V	1729	6.873	0.954	2385.937	2507.877	0.984	127.532

This study was approved by the medical ethics committee of Qilu Hospital of Shandong University. All participants had given the informed written consent prior to their participation.

### Genome DNA extraction and amplicon generation

Total genome DNA was extracted from fecal samples using cetyltrimethylammonium bromide and sodium dodecyl sulfate method. Following concentration and purity monitoring on 1% agarose gels, DNA was diluted with sterile water to 1 ng/μL. The 16S rRNA amplicons covering variable region V4 were amplified using specific primer (16S V4: 515F-806R). PCR reaction was carried out with Phusion^®^ High-Fidelity PCR Master Mix (New England Biolabs, Beverly, MA, USA). The PCR products were quantified with electrophoresis on 2% agarose gel, mixed in equidensity ratios and then purified by Qiagen Gel Extraction Kit (Qiagen, Hilden, Germany).

### Library preparation and sequencing

Library was prepared using TruSeq^®^ DNA PCR-Free Sample Preparation Kit (Illumina, San Diego, CA, USA) followed by quality evaluation on the Qubit@ 2.0 Fluorometer and Agilent Bioanalyzer 2100 system. Library was finally sequenced on the Illumina HiSeq 2500 platform. The 250 bp paired-end reads were produced.

### Paired-end reads assembly and quality control

Paired-end reads were merged using FLASH (V1.2.7) [15]. The raw tags were subjected to quality filtering to select the clean tags with high quality [16] using QIIME (V1.7.0) [17]. The clean tags were then mapped to the reference database (Gold database, http://drive5.com/uchime/uchime_download.html) using UCHIME algorithm [18], followed by removal of the chimera sequences [19] to identify the effective tags.

### Operational taxonomic unit (OTU) clustering and annotation

Sequences were analyzed using Uparse software (Uparse V7.0.1001) [20]. Sequences with similarity of ≥ 97% were assigned to the same OTUs. Representative sequences for each OTU were selected for taxonomic information annotation using Mothur method and the SSUrRNA database [21] in SILVA [22].

### Alpha diversity analysis

The abundance of OTUs was normalized using a standard of sequence number corresponding to the sample with the least sequence. The complexity of species diversity in the sample was analyzed using alpha diversity via seven indices, including Chao1, ACE, Shannon, Simpson, Goods_coverage, Observed_species and PD_whole_tree. The Chao1 and ACE estimator were used to identify the community richness. Shannon and Simpson indices were used to identify community diversity. The Goods_coverage was used to characterize the sequencing depth. PD_whole_tree index was used to identify phylogenetic diversity.

### Beta diversity analysis

The differences of samples in species complexity were evaluated using beta diversity analysis. Beta diversity on unweighted UniFrac was calculated using QIIME to construct unweighted pair-group method with arithmetic means (UPGMA) sample clustering tree. Clustering analysis was performed by principal component analysis (PCA) using ade4 and ggplot2 packages in R software (V2.15.3). Principal coordinate analysis (PCoA) was conducted to get principal coordinates from complex multidimensional data, which were displayed by stat, WGCNA and ggplot2 packages in R software (V2.15.3).

### Differential analysis of intestinal flora at the genera level

OTUs were filtered according to the OTU abundance using QIIME [17] tool, and the OTUs with abundance less than 1/10000 (0.0001) were deleted in this analysis. Then, the remaining OTUs were further screened based on their genus information, and the OTUs that did not annotate to the genus level were removed. Finally, 129 OTUs were obtained. The difference analysis was performed with the edgeR package in R. The OTU screening standard for significant difference was *p* value < 0.05, and false discovery rate (FDR) was used for *p* value adjustment.

## Results

### Sequencing data

After sequencing, the raw reads for all samples ranged from 54,075 to 99,209. The Q20 was more than 98% and Q30 was more than 97% for all samples. After quality control, the effective tags ranged from 80.72 to 95.17%.

### OTU annotation

**Fig. 1:**
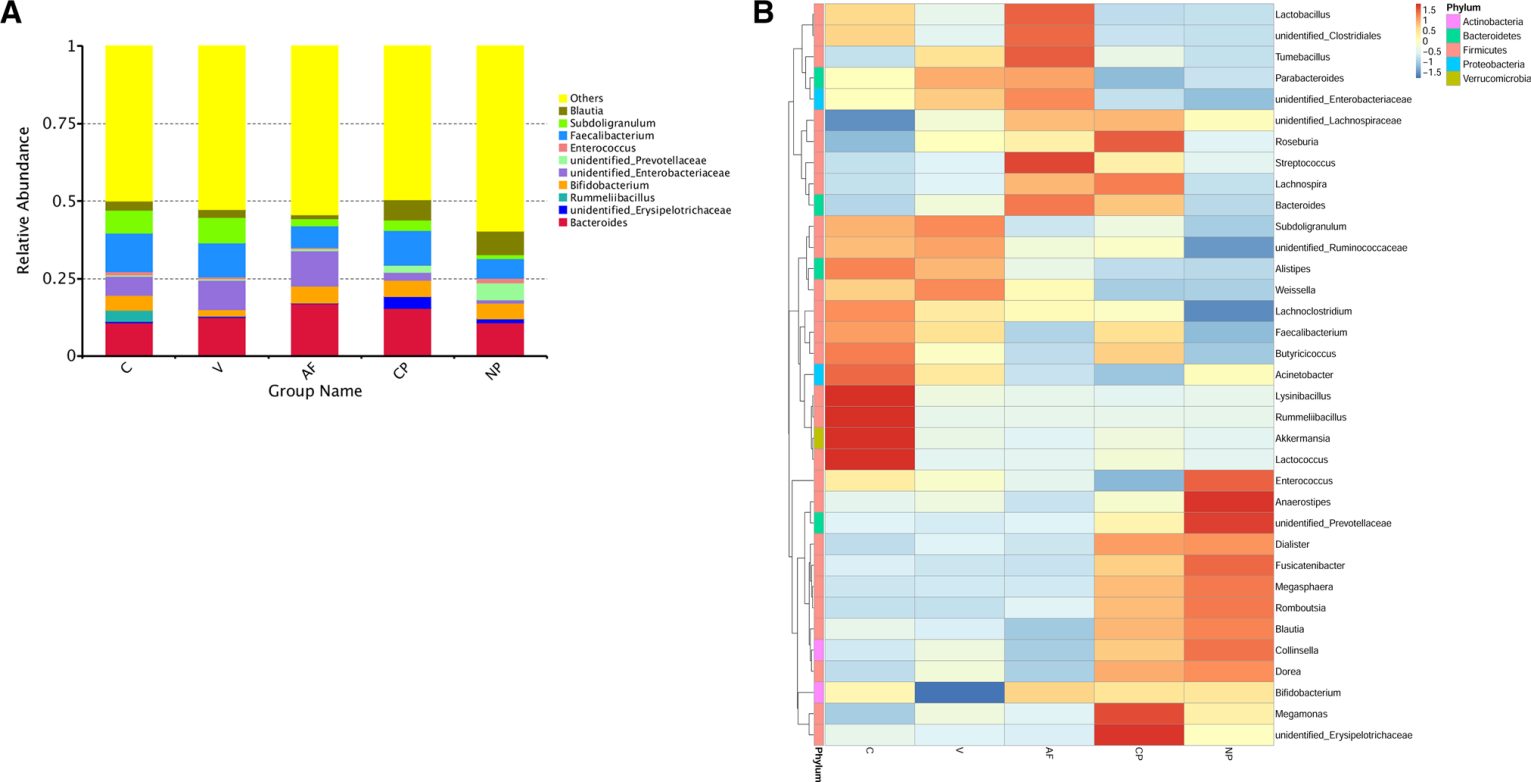
**a** Relative abundance of microflora in genus level (top 10). Others indicated the sum of relative abundance beyond the ten genera. **b** Heatmap of microflora relative abundance

The top 10 maximum abundance of species in each group in genus level is shown in Fig. 1a. *Bacteroides* had the highest relative abundances in V, AF, CP and NP groups, and *Faecalibacterium* had the highest relative abundance in C group. In order to compare the species content in different groups of samples, the top 35 genera were selected for clustering analysis based on the species annotation and abundance information of samples at genus level. As shown in Fig. 1b, in the C group, *Faecalibacterium*, *Bacteroides* and *Subdoligranulum* were the dominant genera; in the V group, *Bacteroides*, *Faecalibacterium* and unidentified *Enterobacteriaceae* were the primary genera; in the AF group, *Bacteroides*, unidentified *Enterobacteriaceae* and *Faecalibacterium* were the dominant genera; in the CP group, *Bacteroides*, *Faecalibacterium* and *Blautia* were of higher abundance; meanwhile, in the NP group, the most abundant sequences were related to *Bacteroides*, *Blautia* and *Faecalibacterium*.

### Alpha diversity analysis

The alpha diversity indexes, including observed species, Chao1, Shannon, Simpson, ACE, Goods_coverage and PD_whole_tree, for five groups are shown in Table 1. Generally, the community richness, community diversity and phylogenetic diversity in AF and V groups were higher than those in the other three groups. The rarefaction curve showed that the curve of each group tended to flatten, indicating that increasing sequencing depths did not help to discover new OTUs, which was in accordance with the sequencing depth index (Goods_coverage) as shown in Table 1.

### Beta diversity analysis

**Fig. 2:**
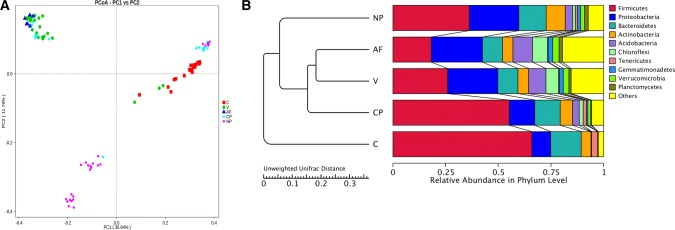
**a** Results of PCoA. The *x*-axis represents one principal component, the *y*-axis represents another principal component and the percentage represents the contribution of the principal component to the sample difference. Each node represents a sample, and the samples from the same group are represented by the same color. **b** UPGMA clustering tree in phylum level

PCoA analysis (unweighted UniFrac distance) showed that the samples in the NP group tended to cluster together, and samples in AF and V groups tended to cluster together (PC1, which explained 36.64% of variation in the community) (Fig. 2a). PCA revealed that the community structure was distinct between NP and the other groups (PC1, which explained 10.33% of variation in the community). Furthermore, the UPGMA clustering tree in phylum level was generated (Fig. 2b). In accordance with PCoA results, AF and V groups were clustered together.

### Differential analysis of intestinal flora at the genera level

**Fig. 3:**
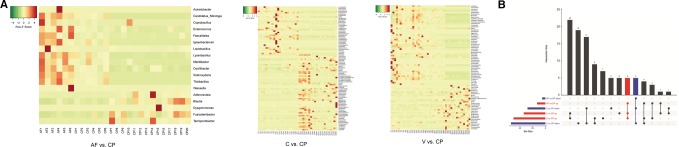
**a** Visualized heatmaps of the differential microbiota (genus level) between acute fatty liver group (AF)/constipation group (C)/excessive vomiting (V group) and normal pregnancy group (CP). Red indicates high relative abundance, and green indicates low relative abundance. **b** Visualization results of intestinal flora differences in the intestinal diseases group (AF, C, V) versus the normal pregnancy group (CP). Red indicates the significantly increased genera in intestinal disease group compared with the normal pregnancy group. Blue indicates the significantly decreased genera in intestinal disease group compared with the normal pregnancy group

The differential microorganisms in digestive diseases (AF, C and V) groups versus CP group, digestive diseases (AF, C and V) groups versus NP group and among digestive diseases groups (AF, C and V) were analyzed. The abundance of 13, 50 and 34 genera was increased in AF, V and C groups compared with CP group, respectively; inversely, a total of 5, 28 and 54 genera were reduced in AF, V and C groups compared with CP group. The heatmaps of three comparison groups are shown in Fig. 3a. In the three comparison groups, the abundance of five common genera, namely *Coprobacillus*, *Acinetobacter*, *Enterococcus*, *Weissella* and *Lysinibacillus*, was increased, while that of five common genera, namely *Terrisporobacter*, *Dysgonomonas*, *Adlercreutzia*, *Fusicatenibacter* and *Blautia*, was identified as decreasing (Fig. 3a, b).

**Fig. 4:**
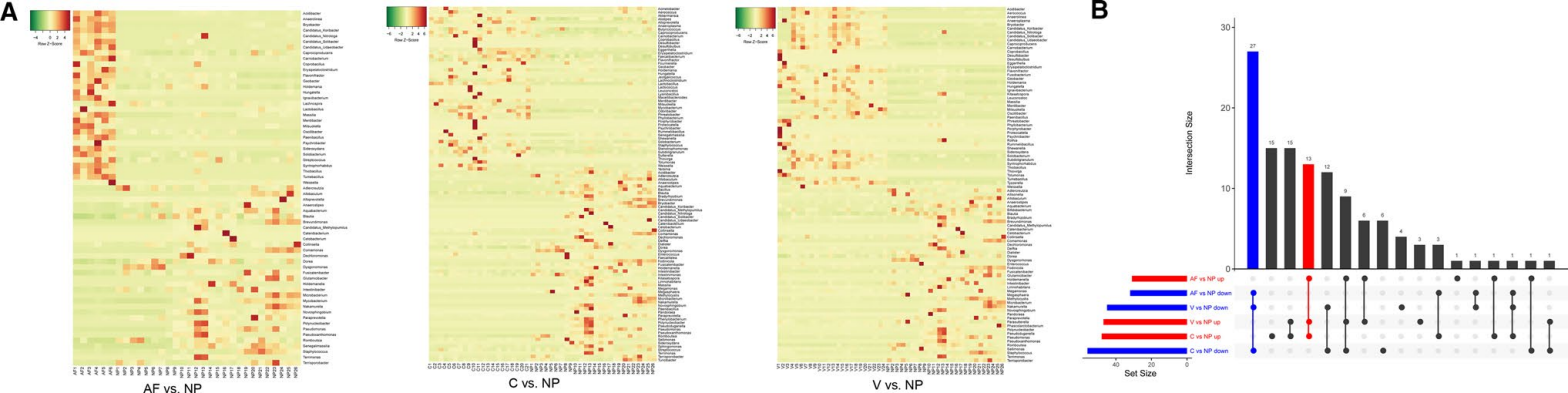
**a** Visualized heatmaps of the differential microbiota (genus level) between acute fatty liver group (AF)/constipation group (C)/excessive vomiting (V group) and non-pregnancy group (NP). Red indicates high relative abundance, and green indicates low relative abundance. **b** Visualization results of intestinal flora differences in the intestinal diseases group (AF, C, V) versus the normal pregnancy group (CP). Red indicates the significantly increased genera in intestinal disease group compared with the non-pregnancy group. Blue indicates the significantly decreased genera in intestinal disease group compared with the non-pregnancy group

There were 31, 47 and 48 genera which were increased among digestive diseases (AF, C and V) groups compared with NP group, respectively; in addition, 32, 45 and 56 genera were decreased in the above three comparison groups. The heatmaps of three comparison groups are shown in Fig. 4a. A total of 13 common genera such as *Carnobacterium*, *Coprobacillus*, *Caproiciproducens* and *Psychrobacter* were observed to be increasing, while 27 common genera such as *Novosphingobium*, *Dorea*, *Brevundimonas* and *Terrisporobacter* were identified to be decreasing (Fig. 4a, b).

**Fig. 5:**
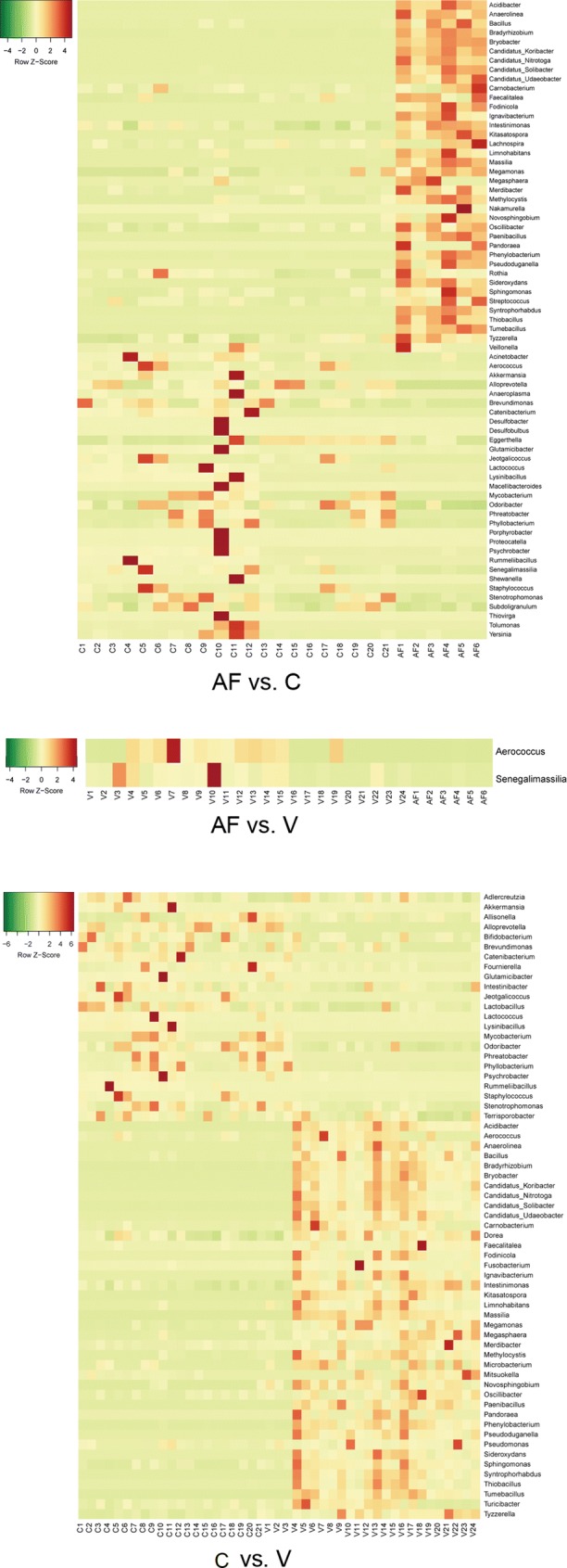
Visualized heatmaps of the differential microbiota (genus level) among acute fatty liver group (AF), constipation group (C) and excessive vomiting (V group). Red indicates high relative abundance, and green indicates low relative abundance

Furthermore, in the AF group, the abundance of 38 genera was increased and 31 genera were decreased compared with C group. The relative abundances of *Aerococcus* and *Senegalimassilia* in AF group were reduced than V group. In the C group, an increased abundance of 23 genera and a decreased abundance of 40 genera were found than V group (Fig. 5).

## Discussion

This study for the first time investigated the gut microbiome composition from pregnant women with digestive diseases by applying 16 s S rRNA gene sequencing. After analysis of the sequencing data, some differential genera were identified, which may serve as biomarkers of digestive diseases during pregnancy.

*Acinetobacter* is a gram-negative coccobacillus, which has emerged from a suspiciously pathogenic organism to an infectious agent important to hospitals around the world [23]. The species commonly cause nosocomial infections, but can also cause urinary tract and soft tissue infections [24]. *Enterococci* are gram-positive bacteria that usually inhabit the alimentary tract of humans [25]. Now enterococci have become common nosocomial pathogens, and enterococcal infections include surgical wound infection, hepatobiliary sepsis, urinary tract infections, bacteraemia and neonatal sepsis endocarditis [26]. *Lysinibacillus*, a ubiquitous gram-positive bacterium, is often regarded as environmental contaminants and rarely associated with human disease [27]. Recently, a case of sepsis due to bacteremia from *Lysinibacillus* and *Paenibacillus* was reported [28]. Given the pathogenicity of the three genera, we speculated that their increase in digestive disease groups compared with CP group may be implicated in the digestive diseases during pregnancy.

*Blautia* are gram-reaction-positive, non-motile bacteria that are obligate anaerobes, and most species of *Blautia* are isolated from the feces of humans and other mammals [29]. The *Blautia coccoides* is the dominant bacterium in the human intestine and accounts for approximately 20–30% of all bacteria in healthy person [30]. It has been reported that decreased relative abundance of the *Blautia* genus in the human gut is associated with malnutrition [31]. The gram-positive genus *Collinsella* contains three recognized species (*Collinsella stercoris*, *C. aerofaciens* and *C. intestinalis*), all of which are isolated from the feces of healthy human [32]. The relative abundance of *Blautia* and *Collinsella* decreased was related to digestive diseases during pregnancy, and *Blautia* and *Collinsella* might serve as novel biomarkers in diagnosis and treatment of digestive diseases.

We also compared the differential microorganisms among various digestive diseases groups. Interestingly, only two differential microorganisms were identified between AF and V groups, suggesting that there was no significant difference in intestinal flora between the AF group and V group. The underlying reason needs to be further explored. Members of *Paenibacillus* have been isolated from water, soil, diseased insect larvae and foods, presenting physiologically diverse characteristics [33]. Some *Paenibacillus* species, such as *Paenibacillus alvei*, *P. polymyxa* and *P. macerans*, are pathogenic, which have been isolated from patients suffering from sickle cell anemia, meningitis and wound infection [34]. In this study, the abundance of *Paenibacillus* was increased between AF versus V group and V versus C group, suggesting an increased expression trend from C to AF. We speculated that increased *Paenibacillus* in intestinal tract may indicate the severity levels of digestive diseases.

In conclusion, our study compared the differences between pregnancy with digestive diseases and normal pregnancy or non-pregnancy. The increase of *Acinetobacter*, *Enterococci* and *Paenibacillus*, and decrease of *Blautia* and *Collinsella* may be associated with the digestive diseases during pregnancy.

## Electronic supplementary material

Below is the link to the electronic supplementary material.

Supplementary material 1 (DOCX 37 kb)
