# Supplementary material for: Changes in intestinal microflora in digestive tract diseases during pregnancy
Source: Arch Gynecol Obstet. 2019 Nov 27;301(1):243–9. doi: 10.1007/s00404-019-05336-0 (PMC7028802; doi:10.1007/s00404-019-05336-0)
Supplement: Supplementary file 1 — Supplementary material 1 (DOCX 37 kb) [file 404_2019_5336_MOESM1_ESM.docx]

Supplementary table 1. The detail information of each sample

| Sample name | gestation period | Gestation weeks + days | Age (years) | Weight (Kg) | Height (cm) | Digestive symptoms | Group |
| --- | --- | --- | --- | --- | --- | --- | --- |
| C1 | 3rd | 25+3 | 23 | 67 | 163 | constipation for two weeks | C^1^ |
| C2 | 3rd | 24+2 | 27 | 78 | 163 | constipation | C |
| C3 | 3rd | 27 | 39 | 80 | 161 | minor constipation | C |
| C4 | 3rd | 38+3 | 36 | 67 | 158 | constipation | C |
| C5 | 2nd | 13+6 | 34 | 67 | 175 | minor constipation | C |
| C6 | 3rd | 28+5 | 28 | 46 | 150 | constipation | C |
| C7 | 3rd | 29+2 | 27 | 62 | 165 | minor constipation | C |
| C8 | 3rd | 30+2 | 29 | 58 | 159 | constipation | C |
| C9 | 2nd | 16+6 | 31 | 64 | 168 | constipation | C |
| C10 | 3rd | 32+3 | 22 | 65 | 158 | Constipation starts from 6^th^ mouth of pregnancy, once every 4-5days. | C |
| C11 | 3rd | 36+2 | 25 | 67 | 163 | constipation and diarrhea | C |
| C12 | 3rd | 38+6 | 25 | 85 | 163 | serious constipation | C |
| C13 | 3rd | 31 | 28 | 60 | 160 | serious constipation | C |
| C14 | 3rd | 38 | 25 | 78 | 162 | constipation for three mouths | C |
| C15 |  | 剖宫产术后12天 | 33 | 70 | 165 | constipation | C |
| C16 | 3rd | 26+4 | 30 | 58 | 158 | constipation | C |
| C17 | 3rd | 28+4 | 34 | 130 | 158 | constipation | C |
| C18 | 3rd | 39+3 | 29 | 71 | 163 | minor constipation | C |
| C19 | 3rd | 38+6 | 31 | 73 | 165 | minor constipation | C |
| C20 | 3rd | 39+6 | 23 | 86 | 161 | fructose treatment for constipation | C |
| C21 | 3rd | 29+1 | 24 | 70 | 170 | constipation | C |
| V1 | 3rd | 26+3 | 34 | 67 | 158 |  | V^2^ |
| V2 | 1st | 11+5 | 28 | 47 | 164 |  | V |
| V3 | 3rd | 40+6 | 27 | 71 | 168 |  | V |
| V4 | 1st | 11+6 | 37 | 49 | 162 |  | V |
| V5 | 1st | 8+2 | 27 | 58 | 162 |  | V |
| V6 | 3rd | 29 | 31 | 85 | 160 | Preeclampsia, abnormal liver function | V |
| V7 | 2nd | 23+2 | 28 | 58 | 161 | Hypertension, acute gastroenteritis, vomiting for one day. | V |
| V8 | 1st | 8+6 | 23 | 55 | 163 |  | V |
| V9 | 1st | 8+5 | 29 | 70 | 165 | IVF_ET^3^ postoperative | V |
| V10 | 1st | 9+6 | 22 | 53 | 155 |  | V |
| V11 | 2nd | 14+ | 26 | 70 | 157 | IVF_ET postoperative | V |
| V12 | 2nd | 18+3 | 25 | 38 | 160 |  | V |
| V13 | 1st | 7+5 | 28 | 51 | 161 |  | V |
| V14 | 2nd | 13+3 | 30 | 62 | 160 |  | V |
| V15 | 2nd | 13+6 | 28 | 53 | 155 |  | V |
| V16 | 3rd | 36+6 | 32 | 68 | 158 | acute gastroenteritis | V |
| V17 | 2nd | 13+ | 26 | 48 | 160 |  | V |
| V18 | 2nd | 15+3 | 33 | 65 | 163 | abnormal lipase liver function, preeclampsia | V |
| V19 | 1st | 11+2 | 29 | 58 | 171 |  | V |
| V20 | 2nd | 15 | 28 | 73 | 160 |  | V |
| V21 | 1st | 11+3 | 29 | 42 | 160 |  | V |
| V22 | 1st | 10+4 | 26 | 48 | 150 |  | V |
| V23 | 1st | 10+ | 41 | 56 | 158 |  | V |
| V24 | 3rd | 29+5 | 29 | 69 | 160 |  | V |
| AF1 | 3rd | 36+4 | 34 | 69 | 158 | hepatitis c, acute pancreatitis | AF^4^ |
| AF2 |  | non-pregnant | 54 | 67 |  | abnormal liver function, mild fatty liver | AF |
| AF3 | 3rd | 40 | 36 | 66.5 | 160 | HBsAg, HBeAg, and HBcAb positive | AF |
| AF4 | 3rd | 38+4 | 30 | 71 | 168 | HBsAg, HBeAb, and HBcAb test positive | AF |
| AF5 | 3rd | 33+2 | 22 | 67 | 173 | SLE^5^, abnormal liver function | AF |
| AF6 | 3rd | 34+3 | 33 | 73 | 164 | abnormal liver function | AF |
| N1 | 3rd | 39+3 | 28 | 97 | 167 |  | CP^6^ |
| N2 | 3rd | 38+2 | 31 | 85 | 161 |  | CP |
| N3 | 3rd | 38+5 | 30 | 76 | 153 |  | CP |
| N4 | 3rd | 39+4 | 29 | 62 | 158 |  | CP |
| N5 | 1st | 13+3 | 35 | 67.5 | 162 |  | CP |
| N6 | 3rd | 35 | 28 | 85 | 165 |  | CP |
| 2 | 3rd | 36+4 | 25 | 72 | 160 |  | CP |
| 3 | 1st | 15 | 34 | 67 | 156 |  | CP |
| 4 | 3rd | 38+5 | 30 | 100 | 168 |  | CP |
| 5 | 3rd | 37 | 34 | 113 | 166 |  | CP |
| 9 | 2nd | 13+5 | 31 | 52.5 | 155 |  | CP |
| E0 | 2nd | 14+4 | 32 | 52 | 158 |  | CP |
| E2 | 2nd | 12+ | 42 | 75.5 | 166 |  | CP |
| E3 | 2nd | 13+2 | 27 | 55 | 150 |  | CP |
| E5 | 3rd | 24+2 | 30 | 70 | 168 |  | CP |
| E6 | 2nd | 18+3 | 31 | 82 | 160 |  | CP |
| E8 | 3rd | 39+2 | 28 | 81 | 158 |  | CP |
| 22 | 3rd | 26+5 | 29 | 77 | 167 |  | CP |
| 25 | 2nd | 16+6 | 34 | 90 | 172 |  | CP |
| 42 | 1st | 9 | 35 | 60 | 168 |  | CP |
| 6 |  | non-pregnant | 37 | 100 | 160 |  | NP^7^ |
| 8 |  | non-pregnant | 28 | 62 | 160 |  | NP |
| E4 |  | non-pregnant | 26 | 56 | 160 |  | NP |
| E7 |  | non-pregnant | 35 | 57 | 160 |  | NP |
| E9 |  | non-pregnant | 25 | 80 | 163 |  | NP |
| 105 |  | non-pregnant | 31 | 45 | 155 |  | NP |
| 108 |  | non-pregnant | 26 | 113 | 160 |  | NP |
| Y |  | non-pregnant | 33 | 58 | 165 |  | NP |
| X2 |  | non-pregnant | 33 | 60 | 157 |  | NP |
| F0 |  | non-pregnant | 33 | 59 | 163 |  | NP |
| 109 |  | non-pregnant | 26 | 50 | 160 |  | NP |
| 106 |  | non-pregnant | 37 | 85 | 163 |  | NP |
| 107 |  | non-pregnant | 37 | 85 | 163 |  | NP |
| 104 |  | non-pregnant | 27 | 52 | 166 |  | NP |
| 103 |  | non-pregnant | 29 | 55 | 164 |  | NP |
| 102 |  | non-pregnant | 33 | 62 | 150 |  | NP |
| 101 |  | non-pregnant | 38 | 62 | 165 |  | NP |
| 31 |  | non-pregnant | 31 | 62 | 160 |  | NP |
| 32 |  | non-pregnant | 26 | 57 | 166 |  | NP |
| 35 |  | non-pregnant | 23 | 65 | 165 |  | NP |
| 36 |  | non-pregnant | 28 | 60 | 160 |  | NP |
| 37 |  | non-pregnant | 30 | 48 | 155 |  | NP |
| 38 |  | non-pregnant | 28 | 53 | 156 |  | NP |
| 39 |  | non-pregnant | 23 | 60 | 163 |  | NP |
| 40 |  | non-pregnant | 35 | 57 | 158 |  | NP |
| 41 |  | non-pregnant | 32 | 50 | 163 |  | NP |

1. C presents constipation group. 2. V presents excessive vomiting group. 3. IVF_ET presents *in vitro* fertilization. 4. AF presents acute fatty liver group. 5. SLE presents systemic lupus erythematosus. 6. CP presents normal pregnancy group. 7. NP presents non-pregnant group.
